# Bimodal Microstructure in an AlZrTi Alloy Prepared by Mechanical Milling and Spark Plasma Sintering

**DOI:** 10.3390/ma13173756

**Published:** 2020-08-25

**Authors:** Orsolya Molnárová, Jan Duchoň, Esther de Prado, Štefan Csáki, Filip Průša, Přemysl Málek

**Affiliations:** 1 Institute of Physics of the Czech Academy of Sciences, Na Slovance 1999/2, 18221 Prague 8, Czech Republic; duchon@fzu.cz (J.D.); prado@fzu.cz (E.d.P.); 2 Institute of Plasma Physics, The Czech Academy of Sciences, Za Slovankou 3, 182 00 Prague 8, Czech Republic; csaki@ipp.cas.cz; 3 Department of Metals and Corrosion Engineering, University of Chemistry and Technology, Prague, Technická 5, 16628 Prague 6, Czech Republic; Filip.Prusa@vscht.cz; 4 Faculty of Mathematics and Physics, Charles University, Ke Karlovu 5, 121 16 Prague 2, Czech Republic; malek@met.mff.cuni.cz

**Keywords:** gas atomization, mechanical milling, spark plasma sintering, microstructure, microhardness

## Abstract

The aim of this study was to prepare a low porosity bulk sample with a fine-grained structure from an AlZrTi alloy. Nanostructured powder particles were prepared by mechanical milling of gas atomized powder. The mechanically milled powder was consolidated using spark plasma sintering technology at 475 °C for 6 min using a pressure of 100 MPa. Sintering led to a low porosity sintered sample with a bimodal microstructure. The sintered sample was revealed to be composed of non-recrystallized grains with an approximate size of about 100 nm encompassed by distinct clusters of coarser, micrometer-sized grains. Whereas the larger grains were found to be lean on second phase particles, a high density of second phase particles was found in the areas of fine grains. The microhardness of the milled powder particles was established to be 163 ± 15 HV0.01, which decreased to a slightly lower value of 137 ± 25 HV0.01 after sintering.

## 1. Introduction

There is a continuous demand for materials with high specific strength. Therefore, the strength improvement of lightweight materials becomes an important topic of research. One of the most studied lightweight materials are the Al-based alloys, where high strength is mostly achieved through precipitation strengthening or grain refinement.

Strengthening through grain refinement can be achieved using severe plastic deformation methods [1]. Alternatively, bulk samples with a fine-grained structure can be prepared by powder metallurgical methods, which consist of the preparation of powders and their compaction into a bulk sample. Techniques like rapid solidification [2] or mechanical milling [3,4] can produce powders with a refined microstructure and enhanced solid solubility of alloying elements. Using a suitable consolidation method, the aforementioned beneficial properties of the powder can be retained also in the bulk sample. A heat treatment applied to those bulk samples can contribute to form a suitable distribution of second phase particles from the supersaturated solid solution that can not only strengthen the material but also help to stabilize the fine-grained microstructure.

The choice of the consolidation method is of great importance. The spark plasma sintering (SPS) combines an applied uniaxial pressure with a heating by low voltage pulsed DC current flowing through the sample [5] and belongs to the most suitable consolidation methods in the Al-based materials (e.g., [6,7]). Compared to other methods, SPS is superior due to the high heating rate, lower sintering temperature and shorter holding time, which help to retain the initial properties of the powder. Several papers dealt with comparison of the effect of different consolidation methods on the microstructure and properties of materials and showed the superiority of the SPS method. Eldesouky et al. [6] showed that samples prepared by SPS from both milled and unmilled Al2124 alloy powders exhibited higher density, microhardness and yield strength compared to samples prepared by hot pressing (HP). Similarly, a more uniform microstructure and higher density were achieved in samples prepared from pure Al and atomized Al5083 powders using SPS compared to HP [8,9]. SPS was also successfully used for the preparation of porosity free Al7075 compacts with extremely high microhardness [10].

The usability of Al alloys at elevated temperatures presents another issue. Exposition of Al alloys to elevated temperatures usually leads to dissolution of strengthening precipitates as the solid solubility limit of most alloying elements used in commercial Al-based alloys increases with increasing temperature [11]. Simultaneously, the enhanced diffusion at elevated temperatures contributes to coarsening of the remaining precipitates [12]. Moreover, the increased temperature can lead to grain coarsening. All these effects result in a strength decrease of Al alloys and a serious limitation of their application field. To retain high strength even at elevated temperatures, aluminum has to be alloyed with appropriate elements, which would form thermally stable second phase particles (resistant to dissolution and coarsening) pinning the grain boundaries and thus preventing grain coarsening. According to the LSW (Lifshitz, Slyozov and Wagner) theory of diffusion driven coarsening of second phase particles at high temperatures [12,13] the alloying elements should have a low solid solubility and low diffusivity in Al not only at room but also at elevated temperatures. Moreover, they should form with Al such intermetallic phases, which have a low lattice mismatch with the Al matrix.

Alloying elements as Zr, Ti, Sc or V belong to the promising ones [14,15]. They have a low equilibrium solid solubility in Al and a very low diffusion coefficient both at room temperature (RT) and elevated temperatures [2,16]. Further, they form trialuminides having a L1_2_ structure (mostly in the metastable state), with a very low mismatch with the Al matrix. Therefore, the second phase particles formed by these elements are resistant to diffusion driven coarsening and can serve as grain boundary and dislocation pinners, thus they delay recovery processes and grain coarsening.

The thermal stability of the Al_3_Zr second phase particles can be further increased by the addition of ternary elements like Ti, V or Hf [14]. Atoms of these elements substitute for Zr atoms in the Al_3_Zr-based particles, reduce their lattice mismatch to the Al matrix and thus also the coarsening rate of these particles. Nearly zero lattice mismatch was observed for the Al_3_(Zr_0,5_Ti_0,5_) particles [15]. It was also shown that the addition of Ti shifts the transformation of the metastable L1_2_ phase to the stable DO_23_ one to higher temperature [17], which further contributed to the strength stability. The maximum Knoop microhardness HK = 150 was found in the Al melt-spun ribbons containing 5 vol. % of metastable Al_3_(Zr_0,5_Ti_0,5_) particles after annealing at 650 K for 1 h. Even a long-term annealing at this temperature for 500 h retained microhardness above the value of HK = 120 [18]. Lower microhardness values were found in the same material prepared by a hot extrusion (at 673 K) of a gas atomized powder [19]. This suggests that hot extrusion is not able to maintain fully the advantages of rapid solidification.

The aim of this study was to verify the possibility of preparation of a fine-grained bulk sample from an AlZrTi alloy where Zr and Ti help to retain the fine microstructure during the processing steps. The samples from this alloy were prepared by a combination of mechanical milling of the gas atomized powder and consolidation by SPS. The microstructure and microhardness of both powder and sintered materials were studied.

## 2. Materials and Methods

The atomized powder of the AlZr2.6Ti0.7 (in wt %) alloy was used as a starting material. The powder was milled for 8 h using a Retsch PM 100 CM planetary ball mill (Retsch GmbH, Haan, Germany). The milling was carried out in an inert Ar atmosphere using a stainless steel vessel and stainless steel balls with a ball-to-powder ratio of 42:1 at 300 revolutions per minute (RPM). The milling process was interrupted for 30 min after every 30 min long milling period in order to restrict heat generation and allow powder cooling. 5 wt % of ethanol was used as a process control agent (PCA) to prevent excessive cold welding of powder particles during milling.

The milled powder was sintered by SPS using an FCT SPS-HP25 (FCT Systeme GmbH, Effelder-Rauenstein, Germany) device in a graphite die. Approximately 3 g of powder were sintered into a cylindrical sample with 20 mm in diameter and 5 mm in height. The powders were sintered at 475 °C for 6 min under uniaxial pressure of 100 MPa. At the beginning of the sintering an initial load of 5 MPa was applied to press the loosely packed powder in the die. Maintaining the pressure, the powder was heated up to 400 °C with a heating rate of 80 °C/min. The sintering temperature of 475 °C was then achieved with a heating rate of 25 °C/min. Simultaneously the pressure was increased from 5 to 100 MPa. The sample remained 6 min at the sintering temperature and then was simultaneously unloaded and cooled down to room temperature. These sintering conditions were chosen as the optimum ones resulting in the lowest porosity and in the best retention of the benefits of powder milling.

For microstructural investigation the powders were mounted into a conductive resin. The microstructure of sintered samples was investigated on thin samples with an area of 5 × 5 mm^2^ cut parallel to the direction of stress applied during SPS. Each sample was polished up to 1 µm of diamond paste, followed by chemical polishing with SiO_2_ suspension (0.25 μm particle size). For transmission electron microscopy (TEM) examination a lamella was cut from a single powder particle with the help of focused ion beam (FIB) using FEI Quanta 3D Dual-Beam SEM/FIB scanning electron microscope. Thin foils for TEM observations were prepared from sintered samples by mechanical thinning to 100 μm thickness followed by electrochemical thinning using a double-jet polisher, Tenupol-5, under 15 V at −15 °C in a solution of 33% nitric acid in methyl alcohol. To ensure the best results, ion polishing was performed using GATAN PIPS Model 691. The samples were ion polished under 4 and 6° at 1.5 keV for 15 min both side.

The morphology and microstructure of samples was investigated using scanning electron microscopy (SEM, FEI Quanta 3D Dual-Beam SEM/FIB scanning electron microscope equipped with field emission cathode) (Thermo Fisher Scientific, Brno, Czech Republic) with secondary electron (SE) and back-scattered electron (BSE) detectors. Finer microstructural details were revealed by TEM and scanning transmission electron microscopy (STEM) investigation, chemical composition of samples was tested by STEM-EDS (energy dispersive spectroscopy). TEM and STEM measurements were carried out using a FEI Tecnai TF20 X-twin microscope operated at 200 kV acceleration voltage (Thermo Fisher Scientific, Brno, Czech Republic).

The phase composition of samples was determined by X-ray techniques. The measurements were performed using an X’Pert Pro PANalytical horizontal powder diffractometer (PANalytical B.V., Almelo, Netherlands) with a Co Kα1,2 X-ray radiation and an X’Celerator detector. The patterns were collected using Bragg–Brentano geometry and subsequently processed by X’Pert HighScore Plus software (2.1b, PANalytical B.V., Almelo, Netherlands) in order to perform phase identification. Quantitative Rietveld refinement was performed in TOPAS V3 software (Bruker AXS, Karlsruhe, Germany) [20], aiming at the determination of the volume-weighted mean crystallite size and wt % of all the identified phases.

Density of the sample was determined by immersion in ethanol (Archimedes method) using the Sartorius YDK03 apparatus at 25 °C.

Vickers microhardness characterizing the mechanical properties was measured both on the powder and sintered samples using a Struers Duramin 2 microhardness tester (Struers, Copenhagen, Denmark) with a load of 98.01 mN (HV0.01).

## 3. Results

**Figure 1:**
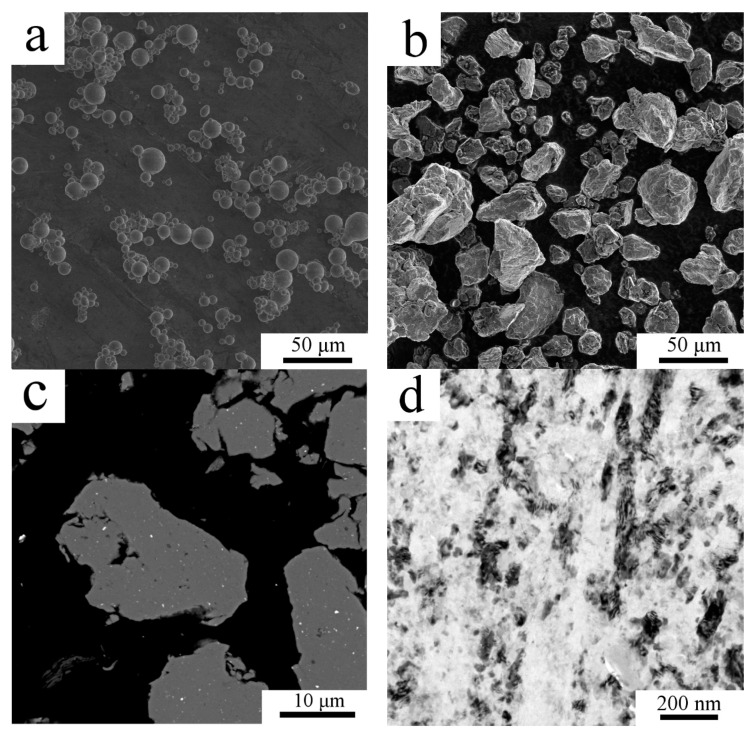

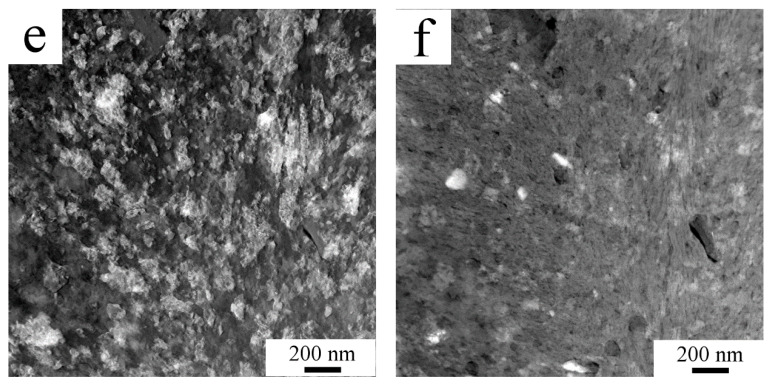
The morphology and microstructure of the AlZrTi powder: the gas atomized powder particles (**a**), milled powder particles showing a wide variety of powder particle size (**b**) and cross section of milled powder particles showing regions with higher atomic number elements (**c**), SEM. The fine grained structure of milled powder particle as revealed by TEM (**d**) and scanning transmission electron microscopy (STEM; **e**,**f**).

The gas atomized powder particles exhibited a mostly spherical shape (Figure 1a) with the mean size of 20 µm. The detailed investigation revealed [21] that the internal microstructure of gas atomized powder particles changes with their size from segregation free microstructure in the smallest particles to microcellular one in larger particles, the typical grain size was 2–3 µm. The repeated fracture and cold welding during milling of the atomized powder led to irregularly shaped powder particles with a very wide size range as presented in Figure 1b by SEM-SE imaging. The average particle size of the milled powder was determined from the SEM-SE images using ImageJ software as 20 ± 11 µm. The large scatter of this mean particle size corresponds to the wide distribution of particle sizes mentioned above. SEM-BSE imaging showed homogeneously distributed regions containing higher atomic number elements in the powder particles (Figure 1c). TEM examination performed on a lamella extracted from one milled powder particle revealed a highly refined microstructure (Figure 1d). Due to the high volume fraction of grain boundaries in the milled powder, there is a high variation in contrast in the sample and the presence of second phase particles is not clearly visible from TEM figures. Therefore, STEM imaging was carried out. Whereas STEM using the high camera length showed strong diffraction contrast (Figure 1e), STEM with a low camera length showed only slight diffraction contrast allowing a higher portion of Z contrast to be detected (Figure 1f). Thus, Figure 1e,f differs in the source of the main component of the contrast but shows the same area under the same sample tilt. Therefore, brighter areas in Figure 1f, which are not bright in Figure 1e are places containing the elements of higher atomic number than the surrounding region. Brighter areas in Figure 1f were found by TEM-EDS to be abundant on Zr and Ti, whereas darker areas were found to contain an increased amount of C and O compared to their surroundings. The presence of these elements is a product of milling using steel vessel and balls and the addition of PCA. The average grain size if the milled powder particles was estimated to be around 70 nm.

**Figure 2:**
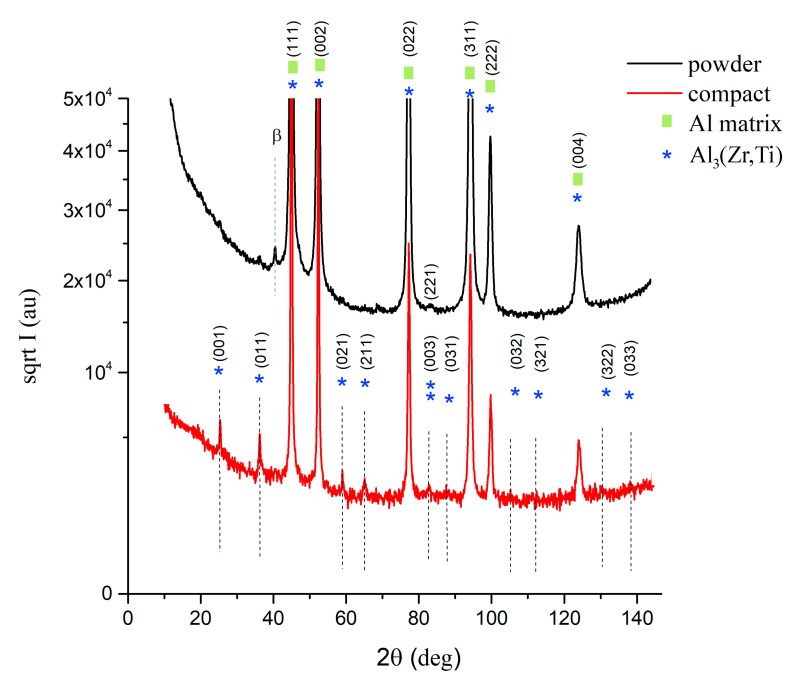
XRD diffractogram of mechanically milled powder and its sintered compact.

The phase composition of the milled powders was investigated using XRD measurement (Figure 2). Although SEM and TEM revealed some areas with elevated concentration of high atomic number elements, XRD showed no presence of second phase particles. Nevertheless, XRD confirmed the fine microstructure of the milled powder with an average crystallite size of 31 nm. For comparison, the crystallite size of atomized powder was 214 nm as stated by the XRD measurement.

SPS of milled powder led to the precipitation of second phase particles as confirmed by XRD measurement. Figure 2 compares the XRD diffractograms of the milled powder and sintered samples (designed as compact in Figure 2). Additional peaks found in the sintered sample correspond to Al_3_(Zr,Ti) particles with the L1_2_ structure. Rietveld analysis showed, that the sample contained 6.4 wt % of these second phase particles.

Sintering by the SPS technique at 475 °C under 100 MPa for 6 min resulted in a bulk compact. The porosity of the sample was determined by the SEM image analysis using the ImageJ program to be 0.76%. The Archimedes method of the density measurement showed a density of 2620 kgm^−3^, which is 95% of the theoretical density of the AlZr2.6Ti0.7 alloy (2749 kgm^−3^).

**Figure 3:**
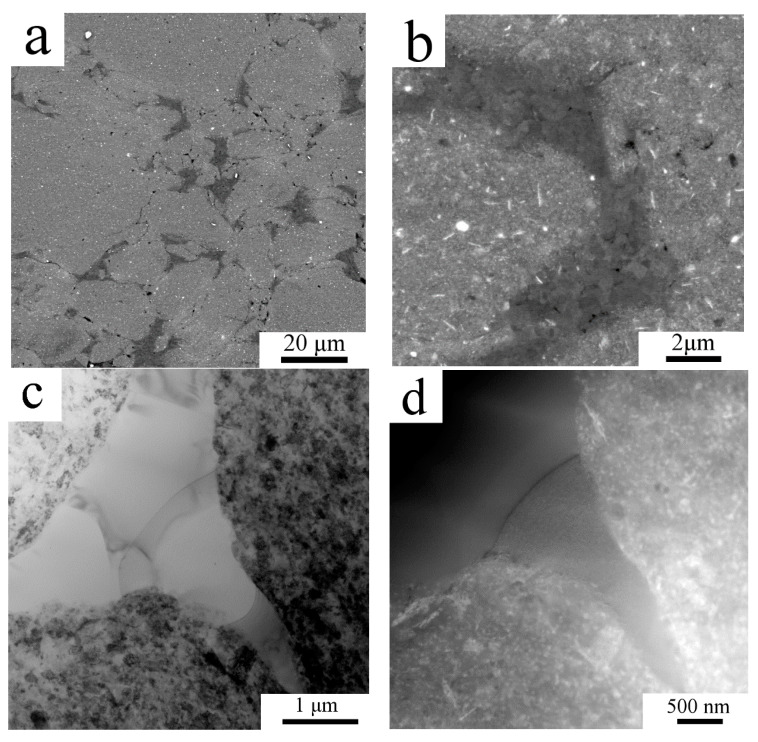

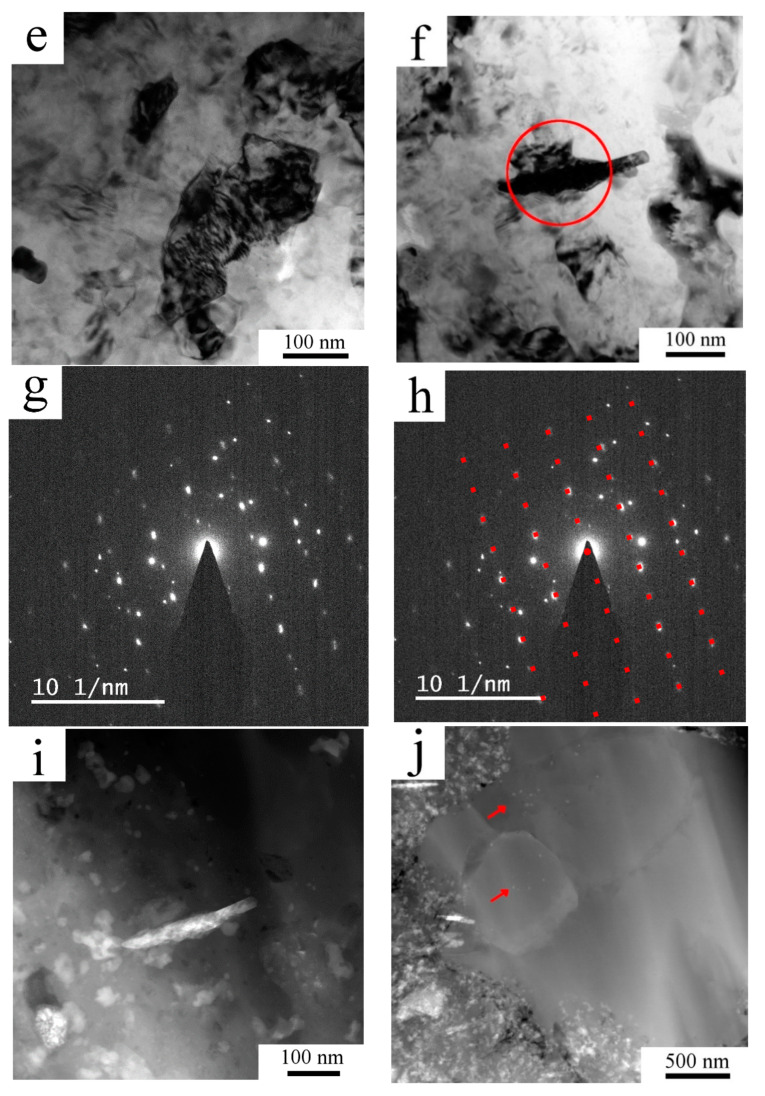
The microstructure of the AlZrTi alloy prepared by milling and spark plasma sintering (SPS): SEM-back-scattered electron (BSE) figure showing regions leaner (darker) and richer (brighter areas) on higher atomic number elements (**a**), higher magnification figure displaying the grains in the darker region through channelling contrast (**b**), TEM figure (**c**) and STEM figure (**d**) revealing the bimodal grain structure along with the distribution of second phase particles, TEM figure showing that most of the nanosized grains remained unrecrystallized (**e**), a prolonged second phase particle (**f**) in the fine grained region was identified as the Al_3_(Zr,Ti) particle by SAED (**g**,**h**) taken from the region denoted by the red circle on (**f**), the same particle examined by STEM using low camera length (**i**), some second phase particles found in the coarse grains using STEM imaging (**j**).

Electron microscopy revealed a heterogeneous microstructure for the sintered sample (Figure 3). SEM-BSE imaging showed two areas with remarkably different contrast (Figure 3a,b). Regions with a lighter contrast were abundant on small second phase particles rich on higher atomic number elements than Al. Regions with a darker contrast lacked second phase particles. Moreover, Figure 3b shows the grains inside the dark contrast regions through channelling contrast, whereas no such feature can be seen in the region of brighter contrast. Image analysis using the ImageJ program revealed that regions leaner on higher atomic number elements than other parts of the sample make up to 10% (area percentage) of the sample.

TEM imaging (Figure 3c) revealed that areas of grains with a size of a few micrometers are surrounded by areas with fine grains with a mean grain size around 100 nm. As on TEM images it is hard to see the second phase particles especially in the region of the fine grains, STEM imaging with small camera length was carried out, which increased the proportion of the atomic number contrast over diffraction contrast. Figure 3d enlarges the right bottom part of Figure 3c allowing us to compare the same region (in the same sample tilt) using TEM and STEM with a small camera length. It shows the high density of second phase particles among the fine grained region. Higher magnification TEM imaging revealed that most of the fine grains remained non-recrystallized (Figure 3e). As some second phase particles were in the given tilt of the TEM sample oriented to the strong diffraction, they appeared dark and could be recognized as a second phase particle among the small grains. One of such a particle is presented in Figure 3f. SAED pattern taken from the area marked by a red circle is presented in Figure 3g, which was evaluated as a diffraction pattern from the <011> zone of the Al_3_(Zr,Ti) particle. Figure 3h shows the overlay of the theoretical diffraction pattern of the <011> zone with the experimental pattern and gives a good correlation. The remaining diffraction spots are from the grains around the oriented second phase particle. STEM imaging with short camera length shows the same second phase particle revealing higher content of the element with a higher atomic number (Figure 3i). Figure 3i also reveals further areas with brighter and darker contrast, the brighter were found to be rich on Zr, Ti or Fe, whereas darker particles contained O. STEM imaging of the regions of coarse grains revealed some second phase particles (red arrows in Figure 3j), which (according to STEM-EDS measurements) contained an elevated amount of Zr and Ti compared to the neighboring matrix, but a thorough study of the second phase particles in the fine and coarse grained regions is out of the scope of this paper and will be reported elsewhere.

**Figure 4:**
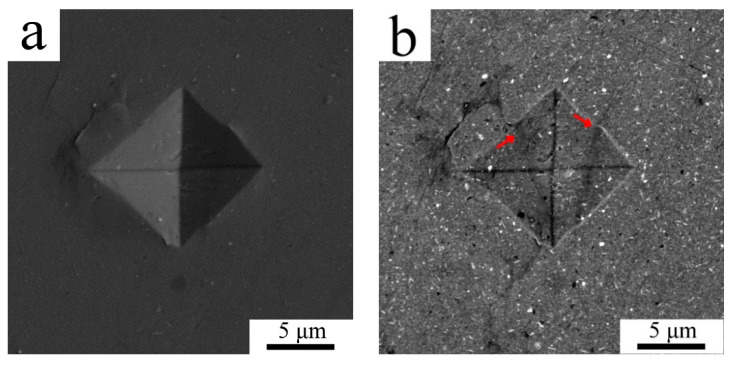

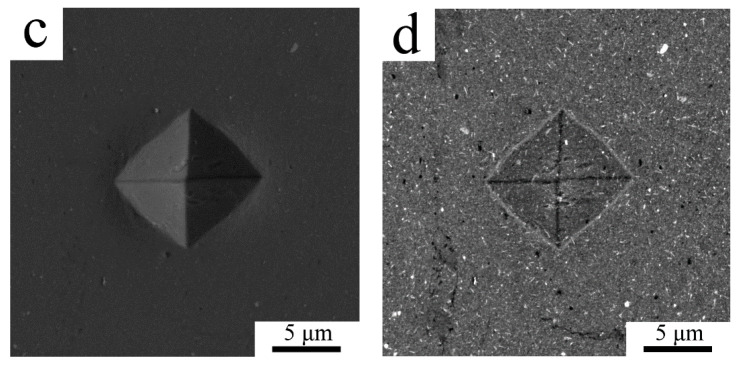
The indents in the sintered sample using SEM-secondary electron (SE) imaging (**a**,**c**) and SEM-BSE contrast (**b**,**d**).

The mechanical properties both of powder and sintered samples were tested by a microhardness measurement. Due to the small size of the milled powder particles the minimum possible load of 98.07 N was applied to make an indent to avoid any deleterious effects. As the diameter of the indents was around 10 µm only the powder particles with the largest polished cross-sections could be tested to diminish the effect of powder edges and the resin itself. The average Vickers microhardness of the milled powder was 163 ± 15 HV0.01. The microhardness of the sintered sample was found to be slightly lower with an average of 137 ± 25 HV0.01, the values ranging from 74.2 up to 161 HV0.01 were measured. The lowest values were measured in areas where the indentation was performed partially in the coarse grained region, an example of such a region is presented in SEM-SE/BSE pair Figure 4a,b. It can be seen in Figure 4b that the indent is crossed by two lines of coarse grained areas (denoted by arrows). The SEM-SE/BSE pair in Figure 4c,d shows a smaller indent inside the fine grained area revealing its higher microhardness.

## 4. Discussion

The mechanically milled AlZrTi powder particles have an irregular shape and exhibit a wide distribution of powder particle size. This morphology is a result of repeated flattening, fracturing and cold welding during impacts of milling balls [4]. Intensive cold welding, characteristic for ductile materials as Al alloys often leads to attachment of milled powder to milling vessel, balls and size increment of powder particles. In agreement with literature data [3,4] we used ethanol as PCA, which helped to prevent excessive cold welding and to retain a relatively fine powder particle size in our material 20 ± 11 µm.

A remarkable grain refinement during mechanical milling is one of the most important results of our research. The grain size of 70 nm determined from TEM examination and crystallite size of 31 nm showed by XRD is a result of severe plastic deformation of powder particles during milling for 8 h with 300 RPM under Ar atmosphere with ball-to-powder ratio of 42:1. A very small grain/crystallite size along with dissolution/alteration of second phase particles and enhanced solid solubility are considered as general features in milled powders [3,4]. Milling of the Al7075 + 1 wt % Zr alloy for 8 h resulted in a crystallite size of 64 nm [10]. Even longer milling of the Al6061 + 2 wt % Zr alloy done for 40 h resulted in a crystallite size of 20–30 nm [22]. Another paper described the effect of 60 h milling on Al2024 and Al7050 alloys with 1 wt % Ti, which led to crystallite sizes around 50 nm [23]. Srinivasarao et al. achieved grain sizes of 30–90 nm in Al-Zr alloys of different Zr content after mechanical alloying of Al with pure Zr or Al_3_Zr powder for 15–300 h with 300 RPM and a ball-to-powder ratio of 10:1–20:1 under Ar atmosphere [24]. The comparison of these data suggests that the influence of individual milling parameters is unambiguous and the final grain size is a result of their suitable combination. Despite large differences in milling conditions the final grain size in all mentioned materials was in the range of tens of nanometers.

Regardless of the relatively low equilibrium solid solubility of Zr and Ti in the Al matrix, mechanical milling extended the solid solubility of alloying elements in the matrix. This was confirmed by XRD showing the absence of second phase particles, although, the atomized powders contained 3.2 wt % of them [21]. Contrary to the findings made by XRD, the SEM and even more detailed TEM inspections revealed areas containing elements with a higher atomic number than Al. However, one has to consider the detection limits of the XRD method. In case of very low content of small second phase particles, or if the particles have a non-ordered structure (caused by severe plastic deformation during milling) XRD is not necessarily able to detect them. As shown in [24], mechanical alloying of Al with pure Zr or Al_3_Zr powder in ethanol as PCA can lead to the formation of the Al_3_Zr phase in L1_2_ or DO_23_ state or other phases with an amount detectable with the XRD technique especially after prolonged milling or at high concentration of Zr.

During the milling process some contamination of milled powders with other elements can occur. Although such processes may be undesirable, they can lead to additional strengthening of powder particles. Partial decomposition of PCA and its integration into the powder already after 1 h of mechanical alloying was shown by thermogravimetry in an Al + TiO_2_ powder mixture [25]. Decomposition of ethanol used as PCA during mechanical alloying of an Al-Zr alloy led to ZrH_2_ or ZrC phase with an amount detectable with the XRD technique [24]. The presence of C and O in the milled powder particle was proved by the EDX analysis in the case of milled Al [26,27]. The milling vessel and milling balls represent another source of possible contamination. Mechanical alloying in the steel vessel using steel balls was shown to lead to implantation of Fe into the milled Al alloy, which then led to formation of Al-Fe phases upon sintering [24]. Nevertheless, the fine and homogeneous dispersion of hardening phases can also help the formation of fine grained structure during milling as it was shown for a milled Al-Ti alloy [6,28]. Similarly, the high strength of the powder in [24] was partially prescribed to the hardening effect of contamination from the milling media. Our experiment revealed a similar behavior, i.e., an increased amount of C, O or Fe as contamination from the PCA and milling environment in the studied powder.

As aluminum is a very reactive element, after exposition to air is readily covered by the oxide layer. The oxide layer on the surface of the milled powder particles was several times claimed to hinder sintering, as it restricts the direct contact of powder particles and hinders diffusion [6,29]. Usually it means that to achieve a dense sample, a strong deformation process, such as extrusion, is needed to produce metal–metal contact through the broken oxide layer. SPS was frequently reported to break the oxide layer of sintered Al powder, allowing powder to sinter and lead to production of a low porosity sample [6,30].

The parameters of SPS used in the present case were selected appropriately and led to a bulk sample with a porosity below 0.76%. The density of the sintered sample was found to be lower than the theoretical value. Considering 1% porosity of the sample, the density of the alloy should be lowered from 2750 to 2720 kgm^−3^, which is still higher than the actually measured value of 2620 kgm^−3^. This discrepancy can be explained by the C and O implantation during the milling process, which occurs normally during mechanical milling, as discussed above. Similar values of relative density as reported here were found also by other authors who used SPS for sintering of similar alloys. Eldesouky et al. [6] reported about a 95% relative density of the sintered sample, whereas 90.5% theoretical density was achieved by Kellog et al. [9] who compacted cryomilled powder. However, this density decrement was not discussed in terms of porosity and implantation of lighter elements.

At the beginning of the SPS process a low pressure is usually applied on the loose powder to rearrange the powder particles, make a denser green sample and increase the number of particle–particle contact. During SPS, densification proceeds during exposition of powders to high pressure and temperature. Usually this is sufficient to make a fully dense sintered sample as it was reported for the sample prepared by gas atomization followed by SPS from the same AlZrTi alloy as presented here [21]. The compact sample sintered from gas atomized powder showed a more uniform microstructure, no bimodal grain size distribution or excessive differences in displacement of second phase particles were observed [21]. In the case of the herein reported milled powder, the SPS led to a non-homogeneous microstructure from the viewpoint of second phase particle distribution and grain size.

Similar bimodal microstructure was reported for an Al5083 alloy prepared by cryomilling and SPS [7]. Whereas the cryomilled powder was reported to be mostly homogeneous and to exhibit a grain size around 28 nm, sintering led to the formation of alternating regions of fine grains with an average size around 50 nm with regions of micrometer sized grains. The coarser grains appeared mainly at the surface of former powder particles and their presence was ascribed to local high temperature regions during sintering, which led to diffusion, melting and severe grain growth [7]. As was shown by Zhou et al. [31] bimodal grain-size distribution can appear also inside the milled powder particles during annealing of the sample. They found, that in the case of cryomilled Al-7.6 Mg (at. %) alloy powder annealing at 250 °C for 1 h led to the formation of regions with fully recrystallized lamellar or equiaxed grains with the size of around 0.3 μm. Coarser grains appeared inside of milled powder particles due to recrystallization or between each initial powder particle due to localized melting and solidification [7,31].

As presented by SEM, TEM and STEM, our milled powder particles already contained distinct and homogeneously distributed regions rich in Zr and Ti. Considering that the coarse grain regions were formed by recovery of given regions of milled powders the second phase particles should be found homogeneously in the sintered sample, without a significant difference of their density in the coarse or fine-grained region. However, this is not the case of the present sintered sample. SEM clearly showed a non-homogeneous distribution of second phase particles, confirmed also by TEM and STEM (see Figure 3). As no coarse second phase particles could be found in the coarse-grained area, the generation of micron-sized grains by recovery of aforementioned regions of milled powder is not probable.

Bimodal microstructure was observed also in a Fe-Al-C sample prepared by mechanical alloying and spark plasma sintering [32]. Nanograined Fe_3_Al with fine precipitates inside were found to be altered by micron sized α-Fe grains, which were also containing some fine precipitates. The authors used powders with a different amount of carbon to prepare alloys with distinct carbon content. The powders with the lowest carbon content were characterized by a uniform microstructure composed of only Fe_3_Al grains as no melting occurred. As the carbon content in powder increased, the melting point of the alloy decreased [32]. Consequently, a partial melting of the powder particles was observed and resulted in a formation of bimodal structure. Similar results were found also by Koizumi et al. in the Fe-23Al-6C alloy [33], by Srinivasarao et al. in Al-Zr alloys prepared by mechanical alloying followed by SPS [24] and by Sasaki et al. in an Al-Fe alloy [34].

Partial melting of powder particles as a source of micron sized grains seems as a reasonable explanation for the present case. The mechanical milling increased the strength of the powder particles, which then could exhibit a lower level of deformability at the beginning and even during sintering [24]. Thus the holes between the powder particles are not necessarily filled through the deformation of individual powder particles. Similar cases, when the applied stress was not high enough to deform the powder particles to fill up the holes between the powder particles, were reported, e.g., in [6,24].

As SPS uses internal heating by Joule heat, places with different resistivity can vary in their exact temperature. Local hot places can be then formed in the sample. These hot places will be obviously at the contact points of the powder particles covered by the oxide layer. At places where the local temperature remains below the melting point, the powder remains in the solid state and densification occurs through rearrangement and (if it is possible) by deformation of powder particles along with rapid mass transport [7,32]. If the local temperature exceeds the melting point of the powder, local melting at the contact of powder particles can occur [32]. Then the melted parts fill the gaps around and the densification of sample follows under the applied external pressure. As the contact area of the powder particles increases the local Joule heating decreases allowing fast heat dissipation within the volume of sintered material.

The microhardness of the gas atomized powder was reported to be 49 HV0.01 [21]. The severe deformation during milling increased this value up to 163 ± 15 HV0.01 due to a common effect of deformation strengthening, remarkable grain refinement and dissolution of alloying elements. Sintering at 475 °C for 6 min then slightly decreased the microhardness of the alloy to 137 ± 25 HV0.01. The relatively high measurement scatter is caused by the non-homogeneity of the microstructure in terms of grain size and second phase particle content. Areas of coarser micron-sized grains were found to exhibit significantly lower microhardness whereas areas of nanosized grains had the highest microhardness values. The drop of microhardness after SPS can be explained by recovery of the fine microstructure of powder. Nevertheless, the average microhardness of the sample sintered from the milled powder is higher than the value reported for sample sintered from the atomized powder (103 HV0.05) or the value measured for hot extruded material (55 HV0.01) [19]. This results especially from a much finer microstructure of the present material.

## 5. Conclusions

The methods of mechanical milling and spark plasma sintering were used for the preparation of the AlZrTi alloy. The results can be summarized as follows:

- The mechanical milling led to powder particles with nanosized grains and introduced C, O and Fe into the powder.
- Spark plasma sintering at 475 °C under 100 MPa for 6 min led to a bulk sample with a porosity of 0.76%.
- The sintered sample exhibited a bimodal microstructure. Areas of extremely fine grains with a mean size of approximately 100 nm altered with areas of coarser, micron-sized grains. The finer grains remained non-recrystallized.
- A high density of second phase particles rich on Zr and Ti was found in the nanosize grained areas, whereas the micron-sized grains contained only a few small second phase particles rich on Zr and Ti. The second phase particles were identified as the metastable Al_3_(Zr,Ti) phase with the L1_2_ structure.
- Partial melting at contact points of original powder particles during SPS is suggested as a reason for the formation of the bimodal heterogeneous microstructure.
- The microhardness of the milled powder was 163 ± 15 HV0.01 and decreased to 137 ± 25 HV0.01 due to the sintering.
